# FDA Facilitated Regulatory Pathways: Visualizing Their Characteristics, Development, and Authorization Timelines

**DOI:** 10.3389/fphar.2017.00161

**Published:** 2017-04-03

**Authors:** Lawrence Liberti, Magda Bujar, Alasdair Breckenridge, Jarno Hoekman, Neil McAuslane, Pieter Stolk, Hubert Leufkens

**Affiliations:** ^1^Centre for Innovation in Regulatory ScienceLondon, UK; ^2^Utrecht Institute for Pharmaceutical Sciences, Utrecht UniversityUtrecht, Netherlands; ^3^School of Life Sciences, University of LiverpoolLiverpool, UK; ^4^Innovation Studies Group, Copernicus Institute for Sustainable Development, Utrecht UniversityUtrecht, Netherlands

**Keywords:** Fast Track, Breakthrough Therapy, Priority Review, Accelerated Approval, facilitated regulatory pathway, FDA, review times

## Abstract

The US Food and Drug Administration (FDA) has four facilitated regulatory pathways (FRPs): Fast Track (FT), Breakthrough Therapy (BTD), Priority Review (PR), and Accelerated Approval (AA). Only PR specifies an expedited review timeline (6 months). We sought to determine to what extent the combination of two or more FRPs influenced development and approval times. We developed a “metro map” to illustrate FRP elements and their influence on review times. We assessed 125 new active substances (approved January 2013 to December 2015) 74 of which used one or more FRPs. For these 74, development times ranged from 1,458 (BTD + PR + AA) to 3,515 days (PR). PR alone had a median approval time of 242 days. The most common combination was FT + PR (median approval 292 days, *n* = 21). The fastest approval times were for PR + FT + BTD + AA (145 days) and PR + BTD + AA (166 days). Our findings support the combination of FRPs for shortening development and review times beyond that provided by PR alone.

## Background

Patients have an expectation of rapid and efficient access to safe and effective, innovative new medicines. This has raised expectations around the speed of the development and regulatory review process. In the US, programs have sought to address these expectations, including the US Food and Drug Administration’s (FDA) Critical Path Initiative which addresses the agency’s strategy to drive innovation in the scientific processes through which medical products are developed, evaluated, and manufactured. The FDA has taken a leadership role in implementing a variety of regulatory pathways that provide sponsors with flexible options to facilitate development, and for the agency to speed the regulatory review process without compromising standards for quality, safety, and efficacy. Four expedited pathways for novel products for serious diseases or unmet medical need are available: Fast Track designation (FT), Breakthrough Therapy designation (BTD), Priority Review designation (PR), and Accelerated Approval pathway (AA). Their characteristics have been well described elsewhere (FDA Guidance for Industry, 2014).

We previously termed these expedited pathways as facilitated regulatory pathways (FRPs): regulatory pathways designed to speed the development, marketing authorization, and patient access to new drugs with a positive benefit–risk balance by providing alternatives to standard product development and regulatory review routes (Liberti et al., 2016). FRPs may increase the level of communication and commitment between the developer and the agency, can give a larger role to effects on surrogate end points, and may move some of the burden of evidence generation from the pre- to the post-authorization phase. Since 2014, more than half of the new molecular entities approved by the FDA used one or more FRPs (Bujar et al., 2015; GAO Report, 2015). However, the extent to which the combined use of these programs affects the time taken in the regulatory review process remains unclear despite growing experience with the programs.

One of the expedited pathways (PR) specifies a shortened review timeline (6 months) and FT and BTD have been designed to encourage interactions between the FDA and sponsors, thereby seeking to shorten development times. Therefore, we sought to determine to what extent these pathways influence development times and whether the combination of two or more FRPs influenced approval times compared to the use of PR alone. We undertook an analysis of products recently approved by FDA to assess the impact of the use of multiple combined FRPs on drug development and approval time. We also developed a simple methodology to illustrate the basic elements of these FRPS and their influence on review times.

## Distinguishing Elements of FDA FRPS

The four FDA programs can be distinguished by several specific characteristics (FDA Guidance for Industry, 2014), including their temporal implementation sequence during development and the nature of the minimally required supportive data: non-clinical evidence of the potential to meet unmet medical need (FT); preliminary human experience suggesting a substantial improvement over available treatments based on a surrogate or intermediate clinical end point (BTD); demonstration of a meaningful therapeutic benefit over available therapies in clinical studies using a surrogate or intermediate end point (AA); and completed clinical trials that have demonstrated a significant improvement in safety and/or efficacy (PR). None of these programs are exclusive and any combination is permissible.

Three of these programs (FT, BTD, AA) have been designed to encourage and expedite development. A product in early development that is granted FT can be supported by early and frequent interactions with reviewers. This support is extended for products granted BTD through organizational commitment from senior agency leadership and the opportunity to receive additional intensive guidance beginning as early as Phase 1. FT and BTD encourage an expedited review by permitting the “rolling review” of sequentially submitted portions of the submission. Products approved via AA are balanced by rigorous post-authorization study commitments. Importantly, PR decreases the statutory review time from 10 to 6 months.

Because outcomes of drug development are often difficult to predict, designations may be rescinded if products do not continue to meet defined criteria upon periodic reassessment. The FT designation may be rescinded at any time if the product no longer meets the qualifying criteria. Not all products assigned BTD will be shown to have substantial improvement over available therapies suggested by preliminary evidence; if clinical benefit is not supported by subsequent data or the non-completion of post-approval trials, the designation may be rescinded. Products with a PR designation must adhere to an integrated post-approval plan (the flexibility of which is determined by the product characteristics, seriousness of the condition and unmet medical need, manufacturing processes, sponsor quality systems, strength of risk-based quality assessment). Products approved via AA are subject to withdrawal if the post-authorization confirmatory trials designed to verify and describe the anticipated effect do not confirm the expected outcomes.

Drug sponsors are required to submit formal requests to use FT and BTD but not for PR (determined by FDA upon start of the review) and AA (assigned by FDA at time of approval). From 2006 to 2014, the FDA Center for Drug Evaluation and Research received about 1,000 requests for FT and BTD (GAO Report, 2015).

## Observations

We analyzed new active substances (NAS) as previously defined (Bujar et al., 2015) that received FDA approval between January 2013 and December 2015. Each was categorized as to the FRP(s) used. Investigational new drug (IND) dates were obtained from public domain data and from the Center for Research Innovation in Biotechnology (CRIB) database^1^. IND submission dates were typically reported in these sources. Where a specific day was not available, the 15th of the month was used. IND dates were found for 68 products in this cohort. Times from IND date to assignment of FT or BTD and to new drug application (NDA) submission were calculated for each instance where dates were available. Time from NDA submission to approval date was calculated based on data obtained from the FDA website. This time in calendar days includes both agency review time and company response time.

**FIGURE 1:**
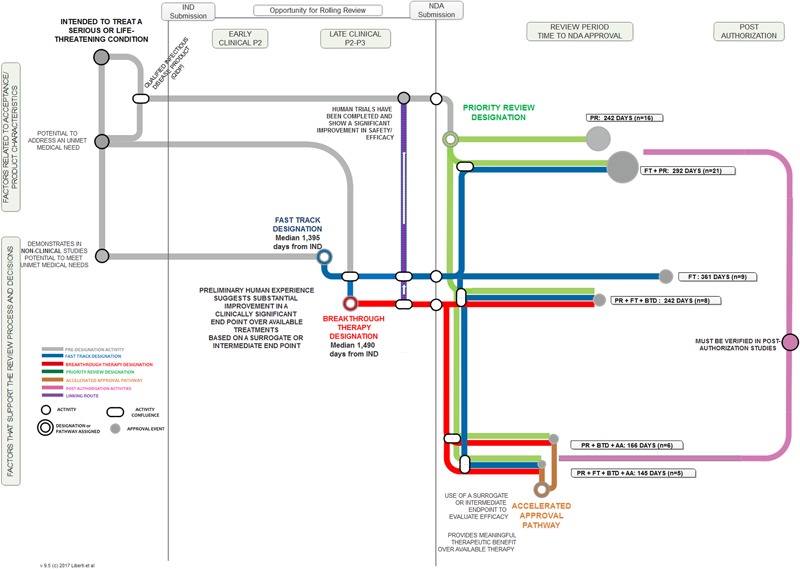
**“Metro Map” analysis of FDA FRPs and influence on median approval times**.

We employed a “metro map” approach to illustrate the relationship between the key aspects of each FRP, the touch points and temporal relationship among them, and the length of the regulatory review times when these programs were employed. **Figure 1** illustrates the key steps for each of the four programs, from pre-IND through to post-authorization. The process begins at the upper left region addressing factors related to acceptance and the product’s characteristics. A product may then follow one of several pre-designation routes to a point at which a designation is assigned (a standard review is always an option and therefore is not illustrated here). The combination of FRP routes result in varying approval times, designated by the tracks in the Review Period sector. The relative length of the review period line corresponds to the median review time; the “node” size at the end of the line reflects the number of products that followed that route.

Among the 125 NAS approved during this period, 74 (59%) used one or more FRP. No products in this cohort used BTD alone, FT + BTD, BTD + AA, or FT + AA + BTD.

**FIGURE 2:**
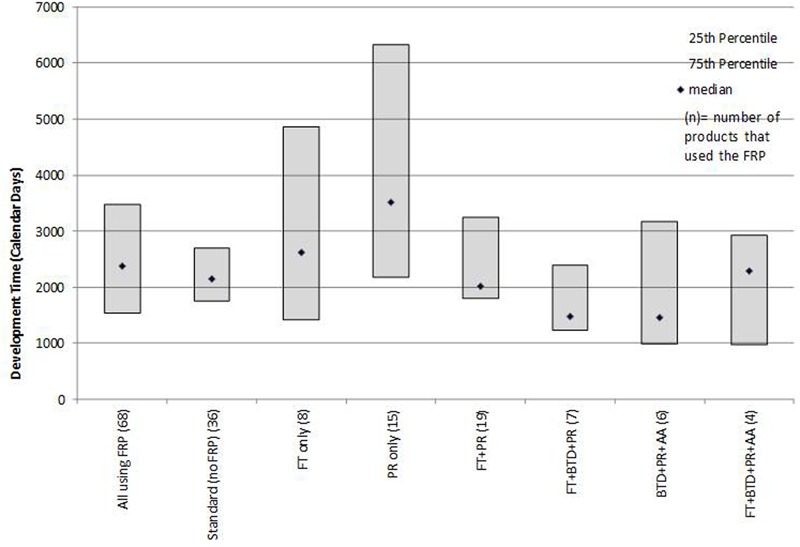
**Median development times (IND to NDA submission) for products that followed one or more FRPs**.

Development times (time from IND to NDA submission) were influenced by the FRP route (**Figure 2**). The median development time for products using any FRP was 2,377 days and for products not using an FRP (standard reviews) was 2,148 days. Products that used BTD + PR + AA had the shortest median development time (1,458 days). By contrast, products that were approved by FT or PR alone had the longest development time (2,620 and 3,515 days, respectively).

Poirier (Poirier and Murphy, 2016) observed that for non-oncology products and vaccines BTD had little impact on development timelines and that AA appeared to influence timelines more than BTD. We observed that for this recent mixed product cohort, the provisions offered by FT and BTD resulted in shorter development times when used in combination with other FRPs. The underlying factors that influence development should be explored, as more products avail themselves of these designations throughout their research phase. The characteristics of the products, influence of unmet medical need, number and outcomes of advice meeting with the agency, nature of the clinical trials or other measures could provide insights into the influence of these FRPs on development program efficiency. Similarly, an analysis of whether products that use an FRP during development stage are more likely to receive first cycle approval could point to what extent FRP use can be a predictor of more efficient regulatory processes.

**FIGURE 3:**
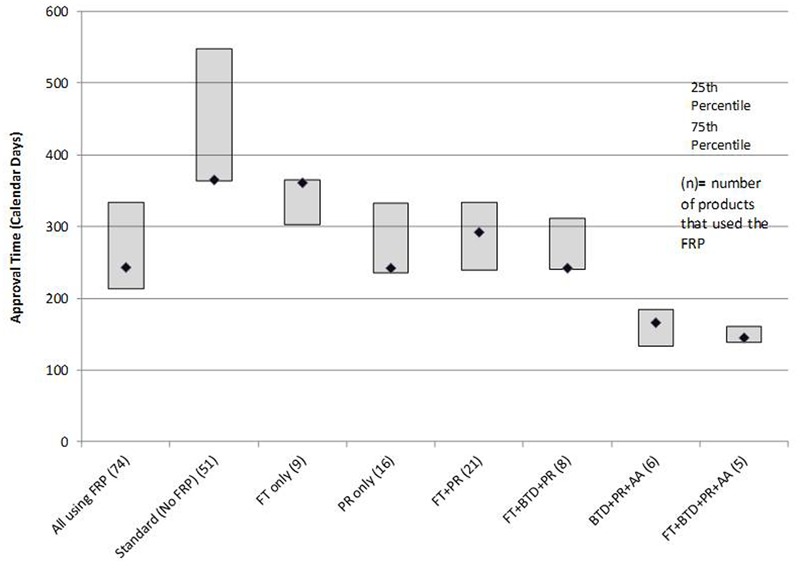
**Median time for FDA approvals for products that followed one or more FRPs**.

In terms of regulatory review, the median approval time for the 74 products that used an FRP was 243 days compared to a median 365 days for the 51 products that did not use any FRP (standard reviews). PR alone had a median review time of 242 days. The most common FRP combination was FT + PR; the median approval time for the 21 products in this category was 292 days. The three fastest review times cohorts were PR + FT + BTD + AA (145 days), PR + BTD + AA (166 days), and PR + FT + BTD (242 days). The median approval times and 25th to 75th percentiles for FRPs used alone or in combination during the analysis time period used by five or more products are presented in **Figure 3**. The median approval times for FRPs used by the remaining products were AA (*n* = 1; 1,034 days), FT + AA (*n* = 1; 304 days), PR + AA (*n* = 2; 328 days), BTD + PR (*n* = 3, 193 days), and FT + AA + PR (*n* = 2; 543 days).

The median approval time for PR + FT + BTD was similar to that of PR alone suggesting that PR is a driver of shortened review time. The cohorts with the shortest review times also received AA. This program gives the agency the flexibility to approve products used for serious or unmet conditions (and with a positive benefit–risk profile) more rapidly on the basis of a surrogate or intermediate efficacy endpoint; expedited access is balanced against post-authorization commitments of continuous assessment of the product’s safety and efficacy linked to disengagement and withdrawal processes if the expected outcomes are not attained.

The use of all four FRPs together was associated with the fastest median approval time (145 days), and this likely reflected the critical importance of the products assessed. All five products that qualified for use of all four FRPs (ibrutinib, idelalisib, nivolumab, osimertinib mesylate, daratumumab) are indicated for the treatment of serious oncologic conditions where there is a high unmet medical need.

When the median development and approval times were taken together, the time from IND to approval was 2,620 days for products that used an FRP and 2,513 days for those that did not use an FRP. Importantly, the shortest overall time from IND to approval was for the cohort of BTD + PR + AA (1,624 days). Combinations of FRPs contributed to faster overall times from IND to approval: FT + BTD + PR (1,720 days); FT + PR (2,308 days); FT + BTD + PR + AA (2,434 days). The longest times were FT alone (2,981 days) and PR alone (3,757 days). The FDA has worked closely with sponsors to manage adherence to post-authorization commitments from FRPs. Where these are not fulfilled, the products may be withdrawn. In a recent example, the FDA approved lutropin alpha for use in infertile hypogonadotropic hypogonadal women under the AA pathway. Subsequently, the sponsor (EMD Serono) requested that FDA withdraw approval of the drug noting that it was not feasible to complete a trial that the company had agreed to at the time of approval; the application was withdrawn in 2016.

BTD has recently been shown to contribute to review times that were faster than target dates defined by Prescription Drug User Fee Act (PDUFA) (Kalesnik-Orszulak et al., 2016). Because BTD was recently instituted (2012) many of the products in this cohort may not have been fully supported by the designation throughout their development cycle; on-going assessments of new approvals will help define the contribution of BTD to the review timeline. Our findings support the value of the combination of FRPs for shortening review times beyond that provided by PR alone. These observations raise questions about the perceived market value of “Priority Review Vouchers (PRV)” wherein an eligible company can use the voucher to have any one of their drugs reviewed under PR.

## Application to Other FRPS

The nature of the data available during a product’s development underpins the selection, sequence, and confluence of FRPs. For example, not all products for serious or unmet medical need qualify for, or may find use of all FDA FRPs. However, the mapping approach presented herein can help illustrate how these programs fit into the overall product development and review process, the interconnections between the designations and pathway, and the relationship of their use to development strategies and approval times.

Similar research can be conducted to provide metrics around the use of novel FRPs in other International Council for Harmonisation of Technical Requirements for Pharmaceuticals for Human Use (ICH) countries [e.g., Conditional Marketing Authorization, Accelerated Assessment, Priority Medicines in the European Medicines Agency (EMA), Early Access to Medicines Scheme in the UK, Sakigake at Pharmaceuticals and Medical Devices Agency (PMDA)], and to assess the outcomes of activities associated with novel adaptive pathways. The utilization of the metro map visualization can serve as a platform to illustrate the requirements, touch points and influence on development, review and approvals times of these FRPs.

The metro map process can also assist in illustrating the routes and timings of specific FRPs often relied upon by maturing regulatory agencies [e.g., the World Health Organization (WHO) prequalification routes, EMA Article 58]. Furthermore, this approach can provide transparency around FRPs being developed and implemented by maturing agencies and regional alignment initiatives around the world (Liberti et al., 2016) and can help identify the different procedures and routes available to enable efficient outcomes through the appropriate application of FRPs.

## Author Contributions

LL, JH, NM, and PS designed the study, designed and presented the results and the metro map, and wrote the manuscript. LL and MB collated and analyzed the data. AB and HL contributed to the manuscript preparation and interpretation of the results.

## Conflict of Interest Statement

The authors declare that the research was conducted in the absence of any commercial or financial relationships that could be construed as a potential conflict of interest.

## Footnotes

1 http://db.crib.wustl.edu
